# General anesthesia for cesarean delivery: Israeli national survey

**DOI:** 10.1186/s44158-025-00257-8

**Published:** 2025-07-01

**Authors:** Daniel Shatalin, Yair Binyamin, Jacob Weinstein, Jana Pirogov, Carolyn F. Weiniger, Sharon Orbach-Zinger, Alexander Ioscovich

**Affiliations:** 1 Department of Anesthesiology, Perioperative Medicine and Pain Treatment, Shaare Zedek Medical Center affiliated with The Hebrew University, Jerusalem, Israel; 2 Department of Anaesthesia, Soroka University Medical Center and the Faculty of Health Sciences, Ben-Gurion University of the Negev, Beer-Sheva, Israel; 3 Department of Anesthesiology, Sheba Medical Center, Tel Aviv University, Ramat Gan, Israel; 4 Division of Anesthesia, Critical Care and Pain, Tel Aviv Sourasky Medical Centre affiliated with Gray Faculty of Medical and Health Sciences, Tel Aviv University, Tel Aviv, Israel; 5 Department of Anesthesiology, Beilinson Hospital, Rabin Medical Centre, Petah Tikva, Israel; 6 Faculty of Medical and Health Sciences, Tel Aviv University, Tel Aviv, Israel

**Keywords:** Cesarean delivery, General anesthesia, Obstetric anesthesia, Survey

## Abstract

**Background:**

Cesarean delivery is one of the most common surgeries performed worldwide. Given the unavoidable use of general anesthesia in some situations, and the potential for complications, we performed a multicenter national survey in order to investigate aspects related to the use of general anesthesia for cesarean delivery.

**Methods:**

This multicenter national survey questionnaire study was performed from October 2020 to March 2021. After Institutional Review Board waiver, we surveyed 25 eligible medical centers with an active obstetric anesthesia unit. The survey covered issues related to general anesthesia: preoperative management, personnel, induction, maintenance and emergence phases of anesthesia, intraoperative and postoperative pain management, protocol use, availability of difficult airway algorithm, and complications that are related to cesarean delivery under general anesthesia.

**Results:**

A total of 113 participants among the 25 medical centers participated in the study. Routine pharmacological aspiration prophylaxis use was reported by 100/113 (88.5%). Administration of opiates during induction before fetal delivery was in 16.8%. We found only 27/113 (23.9%) of respondents ventilate their patients during RSI. Routine use of depth of anesthesia monitoring was reported by 21/113 (18.6%) respondents. Routine postoperative intravenous patient-controlled analgesia (IV-PCA) use with morphine was reported by 6/113 (5.3%) respondents.

**Conclusions:**

In this national survey, we emphasize the importance of the presence of highly qualified anesthesiologic personnel during the surgery, benefit from the use of short-acting opiates during induction, availability of videolaryngoscope, ventilation of the patient during RSI, and availability of institutional difficult airway protocols. We observe underuse of intraoperative anesthesia-depth monitoring and poor postoperative pain control.

**Supplementary Information:**

The online version contains supplementary material available at 10.1186/s44158-025-00257-8.

## Background

Cesarean delivery is one of the most common surgeries performed worldwide [1], and the preferred anesthesia mode is neuraxial anesthesia [2]. Neuraxial anesthesia is associated with less maternal morbidity and mortality and can avoid complications associated with general anesthesia such as aspiration, pneumonia, airway management complications, awareness under general anesthesia, perioperative bleeding, and even potential risk for development of postpartum depression [3–7].

The rate of cesarean delivery has been increasing in the last years up to 50% in some countries [8]. Though a majority of these surgeries are performed under neuraxial anesthesia in some European countries such as Germany [9] and Czech Republic [10], this rate of general anesthesia remains high. Likewise, in Israel, according to a previous national survey questionnaire of obstetric anesthesia units, medical centers report a 20% general anesthesia rate during cesarean deliveries [11].

Given the unavoidable use of general anesthesia in some situations, and the potential for associated complications, we performed a multicenter national survey in order to investigate aspects related to the use of general anesthesia for cesarean delivery.

## Methods

This multicenter national survey questionnaire study was performed from October 2020 to March 2021.

### Ethics approval and consent to participate

The study protocol was approved by the Institutional Review Board (IRB), IRB number 0071-20-SZMC (chairperson: Steinberg Abraham), on May 3, 2020. Written informed consent from each participating physician was not required, as the researchers received anonymized data and did not collect or have access to any identifiable personal information. Verbal consent to participate was obtained. The study was carried out with full cooperation of Israeli Society of Obstetric Anesthesia.

The aim of the study was to review anesthetic management of cesarean delivery under general anesthesia in Israel.

This report is constructed according to the STROBE statement.

The survey questionnaire was constructed and evaluated, and main representatives (director of the obstetric anesthesia units/director of anesthesia department) were validated by three experts, members of the organizing committee of Israeli Society of Obstetric Anesthesia.

The researchers (J. P. and A. I.) contacted the directors of the obstetric anesthesia units or in their absence the director of anesthesia department of all medical centers within the jurisdiction of the Israeli Ministry of Health with an active obstetric anesthesia unit. The main representatives were asked to participate in the study and provide contact information of one attending anesthesiologist and three residents from the same medical center. The data were collected by contacting all participants by researcher, who explained the purpose of the survey and received their verbal consent to participate in the study. The participants were able to complete the questionnaire during the same telephone call or through an electronic questionnaire sent to an e-mail address. The survey was formatted using web service (Google Forms), and the results were stored in Excel file. The questionnaire was anonymous and stored without identification of the participants.

The survey included data on the medical centers and covered issues related to general anesthesia: questions related to preoperative management, personnel in cesarean delivery, induction, maintenance and emergence phases of anesthesia, intraoperative and postoperative pain management, and other issues including protocol use, availability of difficult airway algorithm, and complications that are related to cesarean delivery under general anesthesia in the last 5 years.

The survey comprised 51 separate questions and is presented in Appendix.

To avoid bias, participants were asked to note if their answers were in accordance with departmental practice.

The study outcomes were fasting guidelines, routine use of pharmacological aspiration prophylaxis, staff members in cesarean delivery, ventilation during rapid sequence induction (RSI), videolaryngoscope availability and use, maintenance of anesthesia, planning of postoperative pain management, and other outcomes according to the questionnaire.

Responses were compared for small versus medium/large units.

### Statistical analysis

Data were analyzed using Microsoft Excel Office 2013 and SPSS (IBM) 25.0 (SPSS Inc., Chicago, IL, USA). Data were assessed for normal distribution using the Kolmogorov–Smirnov test and inspection of Q-Q plots. Continuous data will be presented as mean (standard deviation (SD)) and median (interquartile range (IQR)) as appropriate for normal or non-normal data and compared using independent *t*-tests. Categorical variables will be tested using chi-square and presented as counts (percentages).

## Results

Data collection was performed during October 2020–March 2021. A total of 25 eligible medical centers were surveyed, and the response rate was 100%. Medical centers were categorized according to the annual delivery volume: 12 centers had 3000–4999 deliveries, 8 centers had 5000–9999 deliveries, and 5 centers had ≥ 10,000 deliveries.

A total of 113 participants among the 25 medical centers participated in the study, including 21/113 (18.6%) unit directors/director of anesthesia department, 25/113 (22.1%) attending anesthesiologists, and 67/113 (59.3%) residents. There are missing responses of unit directors from three small and two medium medical centers. Missing data about annual delivery volume was obtained from official data of Israeli Ministry of Health (https://www.health.gov.il/English/SearchResults/Pages/GlobalSearch.aspx?k=obstetric).

The median (range) of cesarean delivery rate was 18.5% (10–28%). Annual nonelective cesarean delivery rate was median (range) 47.7% (15–65%). Annual elective and nonelective cesarean delivery under general anesthesia median rate was 3.4% (1–12.5%) and 15.1% (3–27%), respectively.

Data related to preoperative management including fasting guidelines for elective cesarean delivery, guidelines for uncomplicated parturient in labor, and labor with risk factors for cesarean delivery are presented in Table 1. Routine pharmacological aspiration prophylaxis use was reported by 100/113 (88.5%).

**Table 1:** Fasting for elective cesarean delivery, uncomplicated labor and labor with risk for cesarean section, and aspiration prophylaxis medications

		Hospital size		
	Duration of fasting (h)	Small *n* = 54 (100%)	Medium + large *n* = 59 (100%)	*p*-value
Fasting for elective cesarean section				
Fasting				
Fasting (solids)	6	10 (18.52%)	16 (27.12%)	0.28014
	8	37 (68.52%)	42 (71.12%%)	0.75656
	Other	7 (12.96%)	0	.00424
Fasting (light meal)	6	43 (79.63%)	55 (93.22%)	.03318
	8	5 (9.26%)	3 (5.08%)	0.38978
	Other	6 (11.11%)	0	.00854
Fasting (clear fluids)	2	47 (87.04%)	53 (89.83%)	0.64552
	3	1 (1.85%)	3 (5.08%)	0.35238
	4	4 (7.41%)	1 (1.69%)	0.14156
	Other	2 (3.7%)	2 (3.38%)	0.92828
Fasting for uncomplicated labor and labor with risk for cesarean section				
Type of meal/fluid				
Solid meal and all kinds of fluids allowed without limitation	No risk	2 (3.7%)	9 (15.25%)	.03846
	At risk	0 (0%)	6 (10.17%)	.01596
Light meal and all kinds of fluids allowed	No risk	13 (24.07%)	13 (22.03%)	0.79486
	At risk	3 (5.56%)	2 (3.39%)	0.57548
Light meal and clear fluids only	No risk	7 (12.96%)	14 (23.73%)	0.14156
	At risk	7 (12.96%)	5 (8.47%)	0.4413
Meal is prohibited, all kinds of fluids allowed	No risk	5 (9.26%)	2 (3.39%)	0.19706
	At risk	1 (1.85%)	2 (3.39%)	0.61006
Meal is prohibited, clear fluids only	No risk	25 (46.3%)	19 (32.2%)	0.12602
	At risk	26 (48.15%)	30 (50.85%)	0.77182
Total fasting	No	2 (3.7%)	2 (3.39%)	0.92828
	At risk	17 (31.48%)	14 (23.73%)	0.35758
Aspiration prophylaxis medications*				
Medication				
Metoclopramide (Pramin)		25 (46.3%)	32 (54.24%)	0.4009
Sodium citrate		22 (40.74%)	27 (45.77%)	0.5892
Ondansetron (Zofran)		12 (22.22%)	21 (35.6%)	0.11876
Ranitidine (Zantac)		13 (24.07%)	7 (11.86%)	.08914
Famotidine		9 (16.67%)	6 (10.17%)	0.30772
Pantoprazole		5 (9.26%)	10 (16.94%)	0.23014
Esomeprazol (Nexium)		2 (3.7%)	0 (00%)	0.13622

Key: Data presented as frequency data

*Routine use of aspiration prophylaxis medications as a sole agent and in combination with other medications by participants

Elective cesarean delivery under general anesthesia is managed by attending anesthesiologists together with a resident by 67/113 (59.3%) respondents, by residents alone 36/113 (31.9%), and 10/113 (8.8%) by an attending anesthesiologist. No statistically significant differences between small and medium plus large medical centers were found.

The mandatory presence of a neonatologist in a cesarean delivery under general anesthesia was reported by 88/113 (77.9%) participants.

Data related to anesthesia induction phase including airway management, hypnotics, neuromuscular blockers, and opiates are presented in Tables 2 and 3.

**Table 2:** Airway management

	Hospital size		
	Small (*n* = 54)	Medium + large (*n* = 59)	*p*-value
Type of RSI			
• Classical RSI • Modified RSI • No RSI	38 (70.37%) 16 (29.63%) 0	38 (64.41%) 18 (30.51%) 3 (5.08%)	0.50286 0.92034 .09296
Cricoid pressure (Sellick maneuver)			
• Yes, always • Yes, occasionally • No	48 (88.89%) 5 (9.26%) 1 (1.85%)	46 (77.97%) 8 (13.56%) 5 (8.47%)	0.12114 0.47152 0.11642
Ventilation during RSI	7 (12.96%)	20 (33.9%)	.00906
Videolaryngoscopy			
• Always • In case of difficult or suspected difficult airway) • Not available	2 (3.7%) 52 (96.3%) 0	13 (22.03%) 44 (74.58%) 2 (3.39%)	.0041 .00124 0.17068
ETT size			
• 6.5 • 7.0 • 7.5	3 (5.56%) 48 (88.89%) 3 (5.56%)	7 (11.86%) 48 (81.36%) 4 (6.78%)	0.238 0.26272 0.78716
Routine preoxygenation	51 (94.44%)	59 (100%)	.06724
Preoxygenation position			
• Supine • Semi-supine • Other	48 (88.89%) 5 (9.26%) 1 (1.85%)	52 (88.14%) 4 (6.78%) 3 (5.08%)	0.89656 0.62414 0.35238
Preoxygenation type			
• TV for 3 min • 8 VC breaths in 1 min • Pre-oxygenate at least four VC breaths • Other	33 (61.11%) 3 (5.56%) 16 (29.63) 2 (3.7%)	48 (81.35%) 3 (13.56%) 8 (15.38%) 0	.01684 0.9124 0.9124 0.13622
Urine catheter and sterile preparation before intubation	38 (70.37%)	45 (76.27%)	0.4777

Key: Data presented as frequency data

*ETT* endotracheal tube, *RSI* rapid sequence induction, *TV* tidal volume, *VC *vital capacity

**Table 3:** Induction medications

	Hospital size		
	Small (*n* = 54)	Medium + large (*n* = 59)	*p*-value
Induction medications*			
Propofol	45 (83.33%)	36 (61.02%)	.00854
Propofol, ketamine	1 (1.85%)	14 (23.73%)	.00062
Propofol, midazolam	2 (3.7%)	4 (6.77%)	0.4654
Propofol, etomidate	2 (3.7%)	1 (1.69%)	0.50926
Propofol, etomidate, midazolam	1 (1.85%)	0	0.29372
Propofol, ketamine, etomidate	2 (3.7%)	1 (1.69%)	0.50926
Propofol, ketamine, midazolam	1 (1.85%)	3 (5.08%)	0.35238
Neuromuscular blockers			
Succinylcholine	44 (81.48%)	39 (66.10%)	.06432
Rocuronium	10 (18.52%)	20 (33.9%)	.06432
Opiates**			
Remifentanil	0	4	
Fentanyl	5	10	
Total	5 (9.26%)	14 (23.73%)	.04036

Key: Data presented as frequency data

*Routine use of hypnotics and hypnotic combinations

**Routine use of opiates in induction (before fetal delivery)

Routine insertion of orogastric tube was reported by 49/113 (43.4%) respondents. Small size center was 19/54 (32.2%), and medium plus large medical centers were 30/59 (50.8%) (the value of *p* is 0.04236). Routine insertion of temperature probe was reported by 61/113 (54%) respondents. Small size centers were 26/54 (44.1%), and medium plus large centers were 35/59 (59.3%) (the value of *p* is 0.23404).

Regarding the maintenance phase of anesthesia, routine use of inhalational anesthetic was reported by 111/113 (98.8%) respondents (isoflurane 58/111 (51.3%) and sevoflurane 53/111 (46.9%) (the value of *p* is 0.50286)). Participants who change inhalation anesthetic concentration after fetal delivery were 66/111 (59.5%) (the value of *p* is 0.0048), and of them, 33/66 (50%) increase concentration and 33/66 (50%) decrease. Routine use of nitrous oxide was reported by 84/113 (74.3%) respondents, and the concentration used was 50% 50/84 (59.5%), 60% 19/84 (22.6%), and 70% 15/84 (17.9%). When volatile anesthetics are not used, propofol maintenance was reported by 64% (72/113) respondents.

Routine use of depth of anesthesia monitoring was reported by 21/113 (18.6%) respondents: 12/113 (10.6%) used bispectral index (BIS), 7/113 (6.2%) entropy, and 2/113 (1.8%) other monitoring techniques. No statistically significant difference between small and medium plus large centers was found. Use of additional neuromuscular blockers if succinylcholine was used was reported by 52/113 (46%) respondents.

Sugammadex use as routine reversal agent was reported by 59/113 (52.2%) respondents, 48/113 (42.5%) reported use of atropine with neostigmine, and in 6/113 (5.3%) no reverse was used (the value of *p* is 0.13362).

Data related to intraoperative pain management used after neonatal delivery, including opiate and non-opiate medications, are presented in Table 4.

**Table 4:** Intraoperative analgesia — opiates and non-opiates

Medication	Hospital size			
	Small (*n* = 54)	Medium + large (*n* = 59)	*p*-value	Total
Intraoperative analgesia — opiates*				
Fentanyl	33 (61.11%)	13 (22.03%)	<.00001	46 (40.71%)
Morphine	4 (7.41%)	6 (10.17%)	0.60306	10 (8.85%)
Tramadol	0	1 (1.69%)	0.33706	1 (0.88%)
Fentanyl, tramadol	5 (9.26%)	2 (3.39%)	0.19706	7 (6.19%)
Morphine, fentanyl	11 (20.37%)	25 (43.37%)	.01208	36 (31.86%)
Morphine, tramadol	0	4 (6.78%)	.05118	4 (3.54%)
Morphine, fentanyl, remifentanil	0	1 (1.69%%)	0.33706	1 (0.88%)
Morphine, fentanyl, tramadol	1 (1.85%)	6 (10.17%)	.06724	7 (6.19%)
Morphine, fentanyl, Remifentanil, tramadol	0	1 (1.69%)	0.33706	1 (0.88%)
Intraoperative analgesia — non-opiates**				
No	3 (5.56%)	1 (1.69%)	0.267	4 (3.54%)
Ketorolac	2 (3.7%)	1 (1.69%)	0.50926	3 (2.65%)
IV paracetamol	18 (33.33%)	17 (28.81%)	0.60306	35 (30.97%)
Metamizole	2 (3.7%)	5 (8.47%)	0.29372	7 (6.19%)
IV paracetamol, diclofenac (Voltaren)	1 (1.85%)	3 (5.08%)	0.35238	4 (3.54%)
IV paracetamol, ketorolac	6 (11.11%)	1 (1.69%)	.03846	7 (6.19%)
Metamizole, IV paracetamol	14 (25.93%)	11 (18.64%)	0.35238	25 (22.12%)
Metamizole, ketorolac	2 (3.7%)	4 (6.78%)	0.4654	6 (5.31%)
Metamizole, IV paracetamol, ketorolac	5 (9.26%)	7 (11.86%)	0.65272	12 (10.62)
Metamizole, IV paracetamol, diclofenac (Voltaren)	1 (1.85%)	4 (6.78%)	0.20408	5 (4.42%)
Metamizole, IV paracetamol, diclofenac (Voltaren), ketorolac	0	5 (8.47%)	.02852	5 (4.42%)

Key: Data presented as frequency data

*IV* intravenous

*Routine use of intraoperative analgesia opiates after fetal delivery. Combinations

**Routine use of intraoperative analgesia non-opiates. Combinations

Data related to postoperative pain management including peripheral nerve block use are presented in Table 5. Use of infiltration of the surgical wound by local anesthetics was reported by 11/113 (9.4%) respondents. Routine postoperative intravenous patient-controlled analgesia (IV-PCA) use with morphine was reported by 6/113 (5.3%) respondents.

**Table 5:** Postoperative analgesia — opiates and peripheral nerve block

Medication	Hospital size			
	Small (*n* = 54)	Medium + large (*n* = 59)	*p*-value	Total
Postoperative analgesia — opiates				
Pethidine	5 (9.26%)	1 (1.69%)	.07346	6 (5.31%)
Morphine	18 (33.33%)	19 (30.20%)	0.89656	37 (32.74)
Tramadol	0	6 (10.17%)	.01596	6 (5.31%)
Morphine, Percocet	3 (5.56%)	0	.06724	3 (2.65%)
Morphine, pethidine	11 (20.37%)	10 (16.94%)	0.63836	21 (18.58)
Morphine, tramadol	3 (5.56%)	17(28.81%)	.0012	20 (17.7)
Morphine, fentanyl, tramadol	1 (1.85%)	1 (1.69%)	0.95216	2 (1.77%)
Morphine, tramadol, pethidine	9 (16.67%)	5 (8.47%)	0.18684	14 (12.39)
Morphine, pethidine, Percocet	2 (3.7%)	0	0.13622	2 (1.77%)
Morphine, fentanyl, tramadol, pethidine	2 (3.7%)	0	0.13622	2 (1.77%)
Postoperative analgesia — PNB				
No PNB	>41 (75.93%)	49 (83.05%)	0.34722	90 (79.65%)
TAP	11 (20.37%)	10 (16.95%)	0.63836	21 (18.58%)
TAP+QLB	2 (3.7%)	0	0.13622	2 (1.77%)

Routine use of postoperative analgesia opiates. Combinations

*PNB* peripheral nerve block, *TAP* transverse abdominis plane, *QLB* quadratus lumborum block

Reported complications related to cesarean delivery under general anesthesia during the past 5 years included the following: airway and pulmonary complications (aspiration and aspiration pneumonitis, severe bronchospasm, difficult and failed intubation, cannot intubate, and cannot ventilate (CICV)) and intraoperative bleeding. Complications were reported in 15/54 participants from small medical centers and 20/59 from medium plus large centers. (The value of *p* is 0.48392.)

Familiarity of participants with institutional protocol for cesarean delivery under general anesthesia was in 74/113 (65.5%): 39/54 (72.2%) in small and 35/59 (59.3%) in medium plus large medical centers (the chi-square statistic is 2.076; the *p*-value is 0.149635).

A difficult airway algorithm in the operating room was reportedly available for 27/113 (23.9%) participants.

A total of 106/113 (93.8%) participants reported that their answers are according to departmental practice.

## Discussion

This survey provides a current snapshot of anesthetic management of cesarean delivery under general anesthesia in Israel.

According to an updated report by the American Society of Anesthesiologists Task Force and the Society for Obstetric Anesthesia and Perinatology that published practice guidelines [2], it is defined that uncomplicated patients undergoing elective surgery may have clear liquids up to 2 h before induction of anesthesia, a fasting period of 6 to 8 h for solids, and timely administration of nonparticulate antacids, H2-receptor antagonists, and/or metoclopramide for aspiration prophylaxis. We have seen that most participants in the survey provide aspiration prophylaxis, and, overall, there is good uniformity in the issue of fasting to current recommendations. Regarding fasting for elective cesarean delivery, we observe that up to 13% of respondents, mainly from small hospitals, work according to their own practice.

According to our survey, Metoclopramide following Sodium Citrate use as a sole agent or in combination with other medications was the most common medication provided for aspiration prophylaxis in all medical centers. A French survey reported that at least 93% of surveyed participants used at least one aspiration medication and reported widespread use of cimetidine or ranitidine in 62% of units, H_2_ blockers in 28%, and only 4% use of metoclopramide and omeprazole [12]. Another study from Germany shows very widespread use in chemoprophylaxis of acid aspiration syndrome in almost all medical centers. They reported that in 64.2%, there was a preference for the use of combination antacids and H_2_ blockers [9].

Almost 32% of participants reported that there is no senior anesthesiologist in elective cesarean delivery under general anesthesia. In total, 22% reported that a neonatologist is not present at the beginning of the surgery to treat an unexpectedly critically ill newborn, and this is not according to the policy of the Israeli Ministry of Health.

According to the 2015 guidelines of the Obstetrics Anesthesia Association and the Difficult Airway Society, in order to prevent desaturation during RSI, it is recommended to “consider facemask ventilation (*P*_max_ = 20 cmH_2_O).” In spite of these recommendations, we found that less than a third of respondents ventilate their patients during RSI. An additional noteworthy takeaway from the survey is that we would like to see more anesthesiologist use preoxygenation in a semi-supine position with 100% oxygen for at least 3 min in all possible cases, as recommended [13].

The usefulness of videolaryngoscopy in obstetrics is widely adopted, and video laryngoscopes should be immediately available for use as a first-line device [14]. Only 2 (3.4%) respondents reported that the videolaryngoscope was not available. This shows improvement compared to our previous survey that was published in 2019 [11], where this equipment was not available in 16% of units.

Pre-induction opiates have benefits such as blunting of hemodynamic response to laryngoscopy, reducing pain, and the possibility of awareness under anesthesia [15]. A recent meta-analysis has shown that pre-induction opiates such as remifentanil and alfentanil appear to be safe, with no significant effect on Apgar scores or neonatal airway intervention [16]. In our survey, administration of opiates during induction before fetal delivery was reported only in 16%. The data obtained are similar to data from the UK and Australia and New Zealand where rates use of induction opiates in healthy parturients was similar to ours [17, 18].

Because aspiration during extubation is not uncommon and can account for about 50% of all aspiration cases, inserting a large orogastric tube into the stomach after securing airways and removing it before extubation is recommended [19]. In our survey, only 43.36% routinely insert orogastic tube.

For the maintenance phase of anesthesia according to the work of Robins K. and the work of Chin K. J., the admission of sevoflurane ≥ 1.2–1.3% or desflurane ≥ 4.5%, or — in general — volatile anesthetics in doses of 0.7 of MAC, has shown to consistently achieve a mean BIS of < 60, which should prevent awareness [4, 20]. Almost 30% of respondents in our survey administer a low dose of volatile anesthetics at the beginning of the surgery, raising it after the delivery. Recommendations to administer ≤ 0.5 MAC of volatile anesthetics predelivery were common in a few old textbooks [21, 22]. Though this may lead to awareness in up to 1% of cases, these recommendations are less acceptable nowadays. Another close to 30% of respondents reduce the dose of volatile anesthetics in the postdelivery period — perhaps in order to prevent the uterine relaxation effect; however, this is not necessary. When administering less than 1 MAC of volatile anesthetics, the uterus is affected by oxytocin, and there are no clinical signs of uterine relaxation [23].

In order to prevent awareness during anesthesia, the use of BIS, entropy, or other sorts of monitoring of anesthesia depth may be useful, especially for elective or nonurgent cases [20]. According to our survey, only 18.58% of respondents routinely use such monitoring. In the literature, there is no difference between the use of BIS and the use of entropy for cesarean delivery [24]. Our survey found BIS to be more popular; nevertheless, the underuse of anesthesia depth monitoring needs to be changed.

Following the use of  amino-steroidal neuromuscular blocking agents (namely rocuronium), a reversal of neuromuscular blockade may be needed. Data from several studies suggest that sugammadex is superior to neostigmine in terms of speed of recovery and fewer adverse effects. However, the cost of sugammadex still prevents a wider use [25]. In our survey, sugammadex was chosen as a neuromuscular block reversal agent by 52% of respondents, while a combination of neostigmine and atropine was chosen by 42%. The remaining 5% reported no use of block reversal. Our results are comparable to a study conducted in England, where 41.1% of neuromuscular blocks were reversed by sugammadex [15].

In the absence of a neuraxial anesthesia, intraoperative pain management should be carefully planned. In small hospitals, the most common choice is fentanyl as a single agent (61%), followed by combinations of fentanyl and morphine (20.4%) or fentanyl and tramadol (9.3%). However, practitioners from bigger hospitals clearly prefer a fentanyl and morphine combination (43.4%) to fentanyl (22%). Although administration of remifentanil has been previously discussed [26], the use of it in our study group appears to be negligible.

The use of peripheral nerve blocks, namely the ultrasound-guided transversus abdominis plane (TAP) block, has already been proved to be an effective option for postoperative analgesia [27]. In our survey, almost 80% of respondents reported no use of peripheral nerve blocks, while just 18% reported using the TAP block following GA.

Significant maternal pain that is common with general anesthesia for cesarean delivery is associated with less breastfeeding and an increased chance for the development of postpartum depression [28]. Furthermore, chronic pain after poorly managed postoperative pain may have a serious impact on the postoperative course and future maternal quality of life [7]. We believe that more planned intraoperative and postoperative pain management, including the administration of peripheral nerve blocks or IV-PCA, may be one step forward to the solution.

It is well known that there is a very high occurrence of a difficult airway and unexpected difficulty intubating in the obstetric population. Only 24% of the responses reported the presence of a difficult airway algorithm in an operating room, and, in our opinion, it is necessary to improve.

### Limitations

In our survey, the questions were focused on the management of elective cesarean delivery under general anesthesia of healthy parturients. We do not discuss anesthetic management for emergency cesarean delivery of parturients with comorbidities, where the impact of stress, active labor, and underlying pathology may influence anesthetic management decisions. Another potential weakness is that the data was constructed from a questionnaire filled out by a participants who may answer differently from the current practice of their department, but despite this possible weakness, we saw that 93.8% of participants reported that their answers are according to departmental practice.

## Conclusion

In this national survey focused on issues of anesthetic management of cesarean delivery under general anesthesia, we emphasize the importance of the presence of highly qualified anesthesiologic personnel during the surgery; short-acting induction-operation period: benefit from the use of short-acting opiates during induction; improvement in availability of videolaryngoscopes; ventilation of patient during RSI used to prevent hypoxia in case of an unexpected difficult airway; and the availability of institutional difficult airway protocols. We observe underuse of intraoperative anesthesia-depth monitoring and poor postoperative pain control, including peripheral nerve blocks and IV-PCA technique. We hope that, according to this data, medical centers will change their practice on relevant issues.

## Supplementary Information

Additional file 1: Appendix. Questionnaire (Translate from Hebrew).

## Data Availability

No datasets were generated or analysed during the current study.
