# Supplementary material for: General anesthesia for cesarean delivery: Israeli national survey
Source: J Anesth Analg Crit Care. 2025 Jul 1;5:41. doi: 10.1186/s44158-025-00257-8 (PMC12217191; doi:10.1186/s44158-025-00257-8)
Supplement: Supplementary file 1 — Additional file 1: Appendix. Questionnaire (Translate from Hebrew). [file 44158_2025_257_MOESM1_ESM.docx]

**Appendix**

**Questionnaire (Translate from Hebrew)**

1. Hospital name

2. Position

- Obstetric Anesthesia Unit Manager / Chief of department
- Attending Anesthesiologist
- Resident

Hospital Data (Questions for obstetric anesthesia unit manager / Chief of department)

3. What are the number of deliveries in a year?

4. What is the percent of cesarean section deliveries performed in a year?

5. What is the percent of emergent cesarean section deliveries performed in a year?

6. What is the percent of elective cesarean section under general anesthesia performed in a year?

7. What percent of emergent cesarean section under general anesthesia performed in a year?

The following questions relate to preoperative management.

8. What are the fasting guidelines in your hospital for elective cesarean section?

Note – A Parturient without background diseases.

- Solids _____ hrs.
- Light Meal _____ hrs.
- Clear Fluids _____ hrs.

9. What are the fasting guidelines for parturient in labor?

Note – A Parturient without background diseases or risk factors for cesarean section.

- It is allowed to eat (solid foods) and drink without restriction any type of fluids
- It is allowed to eat only light meal and drink any type of fluids
- It is allowed to eat only light meal and drink only clear fluid
- Food is prohibited. It is allowed to drink any type of fluids
- Food is prohibited. It is allowed to drink only clear fluid
- Full fasting should be maintained

10. What are the fasting guidelines for a woman in labor with risk factors for cesarean section?

- It is allowed to eat (solid foods) and drink without restriction any type of fluids
- It is allowed to eat only light meal and drink any type of fluids
- It is allowed to eat only light meal and drink only clear fluid
- Food is prohibited. It is allowed to drink any type of fluids
- Food is prohibited. It is allowed to drink only clear fluid
- Full fasting should be maintained

11. Is there a use of pharmacological aspiration prophylaxis?

Note - If possible, the beginning of the protocol should be as early as possible.

- Yes
- No

12. Please write the name of the aspiration prophylaxis medication.

Note - The question is for those who answered yes to the previous question.

You can mark several answers.

- Metoclopramide (Pramin) - Prokinetic
- Ranitidine (Zantac) - H2 receptor antagonists
- Famotidine - H2 receptor antagonists
- Sodium Citrate - Non-particulate antacid
- Pantoprazole - Proton pump inhibitor
- Ondansetron (Zofran) - Serotonin 5-HT3 receptor antagonist
- Other

13. What is the anesthesia team in an elective cesarean section under general anesthesia?

- Resident Anesthesiologst
- Attending Anesthesiologist
- Attending Anesthesiologist and Resident

14. Presence of a neonatologist in a caesarean section under general anesthesia?

- Always present
- Present only when necessary

The following questions relate to the induction phase in the case of general anesthesia for cesarean section

15. Routine use of cricoid pressure (Sellick Maneuver) during intubation?

- Yes, always
- Occasionally
- No

16. Do you avoid ventilation when necessary while using Sellick Maneuver?

- Yes
- No

17. In case of elective caesarean section under general anesthesia for parturients without background diseases and full fasting, the following will be performed:

- Classical RSI
- Modified RSI
- Induction without RSI

18. Use of Videolaryngoscope.

- In any case
- Only in case of difficult airway or suspected difficult airway
- Does not exist

19. Endotracheal tube size that is usually used

Note - An average woman without history of preeclampsia.

- 7.5
- 7.0
- 6.5
- 6.0
- Other

20. Do you perform preoxygenation routinely?

- Yes
- No

21. In what position is preoxygenation performed?

- Supine
- Semi-supine position
- Other

22. Preoxygenation technique in elective caesarean section.

- Pre-Oxygenate At least 4 VC Breaths
- Pre-oxygenate for 3 minutes, O_2_ 100% (tidal volume for three minutes)
- Eight vital capacity breaths in one minute
- Other

23. In elective surgery, is the insertion of a urinary catheter, clean the surgical site and covering performed before intubation?

- Yes
- No

24. Use of Hypnotics for Induction.

Note - Elective cases, parturients without background diseases. You can mark several answers.

- Propofol
- Ketamine
- Etomidate
- Thiopental
- Midazolam
- Other

25. Use of neuromuscular blocker agents in an induction dose.

Note - Elective case.

- Succinylcholine
- Rocuronium
- Other

26. Use of opiates during induction before fetal delivery.

Note - Elective case.

- No opiates
- Remifentanil
- Fentanyl
- Sufentanil
- Morphine
- Other

27. Routine insertion of Nasogastric Tube.

- Yes
- No

28. Routine insertion of temperature probe

- Yes
- No

The following questions relate to the maintenance phase of anesthesia

29. Use of Volatile Anesthetics.

- Yes
- No

30. Please indicate the type of Volatile Anesthetic.

Note - The question is for those who answered yes to the previous question. Elective case, a parturient without background diseases.

- Isoflurane
- Sevoflurane
- Desflurane
- Other

31. Is there usually a percentage change of volatile anesthetics before and after fetal delivery?

Note - The question is for those who answered yes to the previous question.

- Yes
- No

32. Please indicate the volatile anesthetic concentration used before fetal delivery.

Note - The question is for those who use volatile anesthetics and change concentration before and after fetal delivery.

______%

33. Please indicate the volatile anesthetic concentration used after fetal delivery.

Note - The question is for those who use volatile anesthetics and change concentration before and after fetal delivery.

______%

34. In case you are not using volatile anesthetics, is Propofol maintenance used?

- Yes
- No

35. Is nitrous oxide used?

- Yes
- No

36. Please indicate the percentage of nitrous oxide.

Note - The question is for those who answered yes to the previous question.

______%

37. Please indicate the depth of anesthesia monitoring that in regular use.

- No regular use
- Bispectral Index (BIS)
- Entropy
- Other

38. In case of use of Succinylcholine for induction, is there usually an additional neuromuscular blocker agents used.

Note - If there is no request from the surgeons.

- Yes
- No

The following question relate to extubation

39. Which reversal agent is routinely used?

- Atropine / Neostigmine
- Sugammadex
- Other

The following questions relate to pain management

40. Which of the following medications (Opiate Group) will be routinely given during a cesarean section after fetal delivery.

Note - Opiate Group. You can mark several answers.

- Morphine
- Fentanyl
- Sufentanil
- Remifentanil
- Tramadol
- Other

41. Which of the following medications (Non-Opiate Group) will be routinely given during a cesarean section.

Note - The Non-Opiate Group. You can mark several answers.

- Optalgin
- IV Paracetamol
- PR Paracetamol
- Diclofenac (Voltaren)
- Ketorolac
- Other

42. Which of the opiates is routinely used for pain treatment after caesarean section?

Note - Medications that given in recovery room (PACU). You can mark several answers.

- Morphine
- Fentanyl
- Sufentanyl
- Remifentanyl
- Tramadol
- Pethidine
- Other

43. Is there a routine use Opioid IV Patient-Controlled Analgesia (IV-PCA)?

- Yes
- No

44. Please indicate opiate used in IV-PCA.

Note - The question is for those who answered yes to the previous question.

45. Which of the non-opiate medication is routinely used for pain treatment after caesarean section?

Note - Medications that given in recovery room (PACU). You can mark several answers.

- IV Optalgin
- IV Paracetamol
- PR Paracetamol
- Diclofenac (Voltaren)
- IV Ketarolac
- PO Ibuprofen
- Other

46. Do surgeons usually perform local anesthetic infiltration of the wound?

- Yes
- No

47. In most cases of cesarean section under general anesthesia the following peripheral nerve block is used.

Note - You can mark several answers.

- Usually, no peripheral nerve block use
- Transverse Abdominis Plane (TAP) Block
- Quadratus Lumborum Block
- Other

The following questions address other issues

48. Please note severe complications associated with general anesthesia in cesarean section that have been in your hospital for the past 5 years.

49. If you are familiar with institutional protocol for CS under GA?

- Yes
- No

50. Is there an algorithm for managing a difficult airway in obstetrics in an operating room?

- Yes
- No

51. My answers are according to departmental practice.

- Yes
- No
